# Cardiopulmonary Rehabilitation Improves Respiratory Muscle Function and Functional Capacity in Children with Congenital Heart Disease. A Prospective Cohort Study

**DOI:** 10.3390/ijerph17124328

**Published:** 2020-06-17

**Authors:** Francisco José Ferrer-Sargues, Esteban Peiró-Molina, Pablo Salvador-Coloma, José Ignacio Carrasco Moreno, Ana Cano-Sánchez, María Isabel Vázquez-Arce, Beatriz Insa Albert, Pilar Sepulveda Sanchis, Maria Àngels Cebrià i Iranzo

**Affiliations:** 1 Department of Physiotherapy, Universidad Cardenal Herrera CEU, CEU Universities, 46115 Valencia, Spain; franciscojose.ferrer@uchceu.es (F.J.F.-S.); pablo.salvador@uchceu.es (P.S.-C.); 2 Pediatric Cardiology Section, Hospital Universitari i Politècnic La Fe, 46026 Valencia, Spain; estebanpeiromolina@gmail.com (E.P.-M.); carrasco_jim@hotmail.com (J.I.C.M.); acano20081976@gmail.com (A.C.-S.); beatriz.insa@gmail.com (B.I.A.); 3 Regenerative Medicine and Heart Transplantation Unit, Instituto de Investigación Sanitaria La Fe, 46026 Valencia, Spain; 4 Rehabilitation and Physical Medicine service, Hospital Universitari i Politècnic La Fe, 46026 Valencia, Spain; isabel.vazquez.arce@gmail.com; 5 Universidad San Vicente Mártir, 46001 Valencia, Spain; 6 Department of Physiotherapy, Universitat de València, 46010 Valencia, Spain

**Keywords:** congenital heart disease, cardiopulmonary rehabilitation, cardiac rehabilitation, children, pediatric, respiratory strength, inspiratory pressure, six-minute walking test, physical exercise training

## Abstract

Critical surgical and medical advances have shifted the focus of congenital heart disease (CHD) patients from survival to achievement of a greater health-related quality of life (HRQoL). HRQoL is influenced, amongst other factors, by aerobic capacity and respiratory muscle strength, both of which are reduced in CHD patients. This study evaluates the influence of a cardiopulmonary rehabilitation program (CPRP) on respiratory muscle strength and functional capacity. Fifteen CHD patients, ages 12 to 16, with reduced aerobic capacity in cardiopulmonary exercise testing (CPET) were enrolled in a CPRP involving strength and aerobic training for three months. Measurements for comparison were obtained at the start, end, and six months after the CPRP. A significant improvement of inspiratory muscle strength was evidenced (maximum inspiratory pressure 21 cm H_2_O, 23%, *p* < 0.01). The six-minute walking test showed a statistically and clinically significant rise in walked distance (48 m, *p* < 0.01) and a reduction in muscle fatigue (1.7 out of 10 points, *p* = 0.017). These results suggest CPRP could potentially improve respiratory muscle function and functional capacity, with lasting results, in children with congenital heart disease, but additional clinical trials must be conducted to confirm this finding.

## 1. Introduction

Congenital heart disease (CHD) is the most frequent form of congenital malformations, enclosing a third of the congenital anomalies detected during the prenatal and childhood periods [1], with a global estimated incidence of 8–10‰ of live births [2]. CHD represents a considerable burden both in developed and undeveloped countries [3], and it has substantial economic impact on all health systems [4].

In the last three decades, critical surgical and medical advances have considerably increased the survival rates of CHD patients [5], remarkably increasing the number of patients with moderate and severe forms of CHD that reach adulthood [6]. This “paradigm shift” has repositioned the focus of interventions, previously centered on increasing survival, and now attempting to achieve a higher health related quality of life (HRQoL) [7,8].

CHD patients have impaired aerobic capacity and pulmonary function [9] compared to paired age and gender healthy controls. These impairments have been widely associated with increased morbidity and mortality [10], and they have been linked to severity of CHD, number of surgical procedures, surgical complications, and low body mass index (BMI). This last risk factor points to a relationship, previously described in adult heart failure and CHD, with a lack of respiratory muscle strength [9,11]. Focused on these findings, and their potential improvement, several therapeutic approaches have been explored in the last decade. Physical activity has proven to be beneficial in improving aforementioned capacities [12], and subsequent research on the topic of cardiopulmonary rehabilitation specific for CHD has flourished [13,14,15].

There is increasingly strong evidence suggesting amelioration of aerobic capacities and HRQoL following cardiopulmonary rehabilitation programs in children with CHD [14,15,16], but there is scarce information about the effects of this intervention on respiratory muscle function, despite the acceptance of its relation as a risk factor of poor pulmonary function and its association with HRQoL in children [9].

This study evaluates the effect of a cardiopulmonary rehabilitation program, including respiratory muscle training, on respiratory muscle function, functional capacity, and exercise subjective perception of children with congenital heart disease.

## 2. Material and Methods

### 2.1. Trial Design and Ethics

A single center prospective cohort study was designed and conducted in compliance with the Good Clinical Practices protocol and Declaration of Helsinki principles. It was approved by the Hospital Universitari i Politècnic La Fe Ethics Committee (registration number: IIS La Fe - 2017/0506), on 4 December 2017. The patient information sheet was explained, and all subjects and their legal guardians gave their informed consent for inclusion before they participated in the study.

### 2.2. Participants

All patients scheduled for cardiopulmonary exercise tests performed in the pediatric exercise physiology laboratory of Hospital Universitari i Politècnic La Fe between December 2017 and January 2020 were screened as potential candidates for the study.

From all patients screened, the inclusion criteria were defined as (1) age between 10 and 16 years; (2) height greater than 135 cm; (3) presence of a significant congenital heart abnormality; (4) abnormal exercise capacity, defined as a peak oxygen consumption and/or peak oxygen consumption of less than 80% of the predicted values for age, gender, and height; (5) willingness to be part of the study and participation commitment from the patients and their parents or legal guardians; and (6) signature of the informed consent after thorough program and study information. We excluded any patients presenting (1) personal history of documented life-threatening arrhythmias, (2) inability or contraindication to perform required physical activity, (3) significant depression of left or right ventricle function (subjective or left ventricular ejection fraction < 54%), and (4) hypotensive response to exercise in cardiopulmonary exercise testing (CPET).

### 2.3. Safety Considerations

All measurements, evaluations, and interventions in the context of the present study were performed in a safe environment with an emergency resuscitation trolley equipped with a defibrillator, manual ventilation devices, and CPR medications needed. Patient’s vitals were always continuously monitored during measurements and rehabilitation and continuous ECG was registered and real-time visualized by a pediatric cardiologist during training sessions. Real time ECG registry and visualization was accomplished using Nuubo^®^ wearable ECG technology (Nuubo, 28043, Madrid, Spain).

### 2.4. Measurements

#### 2.4.1. Anthropometric Characteristics

Before medical anamnesis at the exercise physiology laboratory, anthropometric measurements were collected in all participants, including weight (kg) and body fat percentage using an electronic scale TANITA BC-545N (TANITA Corp, Illinois 60005, USA), height (cm) using a manual scale (SECA, Hamburg 22089, Germany), and skinfolds (triceps, biceps, subscapular, and suprailiac) with a Holtain Tanner/Whitehouse skinfold caliper (Holtain Ltd. Crosswell, Crymych, Pembs., SA41 3UF, UK.). BMI (kg/m^2^) was calculated by dividing weight by the square of height in meters. Standard deviation (SD) scores were calculated for weight, height, and BMI according to the Spanish population standards recently published by Carrascosa et al. [17].

#### 2.4.2. Baseline Lung Function

Spirometry was performed using a Cortex Metalyzer 3B (CORTEX Medical, Leipzig, Germany) gas analyzer, and consisted of a flow volume loop recording the forced expiratory volume in 1 second (FEV_1_, L), the forced vital capacity (FVC, L), and the FEV_1_/FVC ratio (%). The test was repeated at least three times to ensure reproducibility, and it was deemed valid after maximality criteria was fulfilled [18] (generally a difference in values less than 5%). For percent values, we used the prediction equations by Zapletal et al. [19].

#### 2.4.3. Respiratory Muscle Function

Maximum Static Inspiratory (MIP) and Maximum Static Expiratory (MEP) pressures were measured in sitting position, using a MicroRPM device (Carefusion, VYAIRE MEDICAL, UK.). In order to minimize subjects’ training and motivation impact on the results, careful explanation of the test was carried out, subjects were vigorously encouraged, and MIP and MEP measurements were repeated until registration of three acceptable and reproducible measurements (difference < 10%), with one minute rests between them, and the highest value was registered [20,21]. Predicted values were estimated using the equation proposed by Heinzmann et al. [22] Maximal voluntary ventilation (MVV, L/min) was estimated from pulmonary function, using the formula FEV1*35 [23].

#### 2.4.4. Functional Capacity

Assessment of functional capacity was carried out using the six minutes walking test (6MWT), selected by its reproducibility, agreement, and criterion validity shown in pediatric patients with this particular group of disease [24]. A pulse oximeter, a stopwatch, two cones to mark the end of the route, a writer Borg scale, and a blood pressure monitor were employed. We registered the maximum distance in meters covered along a 30 m corridor for six minutes. Standardized phrases of encouragement were played every minute of the test. Two trials with a 30-min rest between them were performed after thorough explanation of the procedure in order to minimize the impact of the training effect on the results, and the highest walking distance was used for analysis. To further avoid the effect of motivational variation, the physiotherapist responsible for monitoring continuously inspirited the subject to keep pace and interest. Peripheral oxygen saturation (%), heart rate (HR, bpm), and dyspnea-muscle fatigue (CR-10) were recorded at rest and the end of the test. For predicted values, we used the equation proposed by Geiger et al. [25].

All measurements were collected at the beginning of the program (T1), after all programmed sessions (T2), and six months after conclusion (T3), and all the tests were performed at the same time of day. We also collected data regarding regular physical activity before and during the program to account for possible biases.

### 2.5. Intervention

All subjects were included in a tertiary center pediatric cardiopulmonary rehabilitation program (CPRP) (IMPROVE project). The IMPROVE intervention was designed following the American College of Sports Medicine (ACSM) Guidelines for exercise prescription, considering the FITT (Frequency, Intensity, Type, and Time) principles for cardiac patients and adjusting them to the pediatric population [26]. Frequency was set to two times per week. Intensity was adjusted from CPET parameters, regulating endurance training to achieve a HR near VT1 at the beginning of the program, and progressively moving towards VT2 or a maximal HR of 75% of peak HR. Training was devised following the Skinner and McLellan model [27]. The type of intervention included endurance and resistance training. The endurance exercises were conducted in the different modalities of the continuous training (uniform and variant pace). Each training session lasted for 70 min, and a total of 24 supervised sessions were performed in harmony with the recommendations of previous studies [13].

All subjects were monitored during the session. Peripheral oxygen saturation, heart rate, and real time ECG were acquired continuously. Blood pressure was measured at the beginning and the end of sessions with an Omron M6 Comfort Blood Pressure Monitor (Omron Healthcare Europe B.V, Hoofddorp, The Netherlands). Patients’ perceived exertion was registered using a Borg CR-10 scale at the beginning, after each training phase, and at the end of each session. Training was always led by two experienced physiotherapists, and personally supervised by a pediatric cardiologist.

Training sessions were structured in five different phases: (1) *Warm-up phase (5 min).* This phase included diaphragmatic breathing, articular mobility exercises, and a light walk. (2) *Endurance-training phase (20 min).* Aerobic training was carried out using a treadmill (BH Fitness) and a static bicycle (BH Rhyno Max H491), including two minutes of warm-up, sixteen minutes of continuous training, and another two minutes of cool-down, in line with recommendations of endurance training for children with CHD [28]. We chose the uniform or variant pace based on the progression of the patients during the intervention. Intensity was set according to previously explained FITT parameters. (3) *Resistance-training phase (20 min)*. According to the session, the subjects completed three series of four exercises. During the first sessions, training was done with light and medium resistance bands, emphasizing the analytical workout of principal muscles (deltoids, biceps brachii, triceps brachii, abdominals, trunk extensors, quadriceps, hamstrings, and calves). Since session nine, we progressed into a functional training, using gymnastics equipment as dumbbells, medicine balls, steps, and plyometric workout. They made 10–15 repetitions of each exercise, with a 20 second rest. As a motivational complement, the last sessions incorporated virtual reality games. (4) *Respiratory-training phase (20 min)*. As a final phase of muscular training, a specific respiratory muscle workout was conducted using the Threshold^®^ Inspiratory Muscle Trainer device (Respironics, NJ 07054, USA), adjusting the workload to a minimum of 30% of subject MIP [29]. Range workload of the IMT device was between 9 to 41 cmH_2_O. With all the children sitting comfortably, the protocol contained 21 min of training divided into seven series, with two minutes of work and one of rest between series. During this training, a physiotherapist reeducated the ventilatory pattern, avoiding the use of accessory respiratory musculature and the increase of respiratory rate and/or tidal volume. To ensure that each patient was training with an appropriate workload, two intermediate-study MIP measurements were taken (weeks 4 and 8). (5) *Cool-down phase (5 min).* It included a light walk and body stretching, especially upper and lower limbs, in order to normalize vital signs and minimize perceived exertion at the end of the session.

In addition to supervised training sessions, children were encouraged to stay active throughout the week, participating in physical education at school and non-competitive games. Regarding respiratory training, as the protocol had to be trained three times a week, two of them were performed during the sessions, while the other one was carried out at home [30]. Patients received a guide and registered session completion and incidents.

### 2.6. Statistical Analysis

All data preparation, exploration, analysis, and plotting were performed using Python programming language data science open-source libraries including: (1) Numpy (Copyright © 2020–2020, NumPy Developers), (2) Pandas (Copyright (c) 2008–2011, AQR Capital Management, LLC, Lambda Foundry, Inc. and PyData Development Team), (3) Matplotlib (Copyright (c) 2012–2013 Matplotlib Development Team), (4) Seaborn (Copyright (c) 2012–2020, Michael L. Waskom), (5) Scypy (Copyright© 2020–2019 SciPy Developers), and (6) StatsModel (Copyright© 2020–2018 StatsModel Developers). Distribution of quantitative variables was strongly tested for normality before inferential analysis by performing Shapiro–Wilk, D’Agostino K^2, and Anderson–Darling tests. Bivariate association was investigated using related and non-related one sample t-test in case of normally distributed variables, and Mann Whitney U and Wilcoxon signed-rank test for non-distributed variables depending on data pairing. Bonferroni correction was applied to account for multiple measurement comparisons potential alpha error. Categorical bivariate association was studied using Fisher’s exact test. Data are presented as mean values (SD) or median (IQR) in non-normally distributed variables. A *p*-value < 0.05 was considered statistically significant. Sample size calculation for paired mean differences was calculated assuming a level of significance of 0.05, a statistical power of 70%, and an effect size of 0.6, resulting in a minimum sample size of 15 patients.

## 3. Results

### 3.1. Population

A total number of 353 subjects were screened at the exercise physiology laboratory. Twenty-eight patients fulfilled clinical criteria and were contacted. Participation in the study was declined by 13 subjects. The main reasons not to participate were geographical limitations and the time-consuming exigencies of the program, respectively. All demographic characteristics of the screened patients were documented. Amongst the patients that fulfilled clinical criteria, there were no significant differences between the ones that accepted and rejected participation in terms of gender, age, or anthropometric characteristics.

A total of 15 patients were enrolled (mean age 14.4 years, 60% male). All patients had undergone corrective surgery or heart transplant. Patients diagnoses were Tetralogy of Fallot (6), Heart transplantation (3), D-Transposition of great arteries (2), Pulmonary Atresia with intact ventricular septum (1), Pulmonary atresia + VSD (1), repaired VSD (1), and repaired Taussig–Bing anomaly (1). All patients reported two mandatory sessions per week of light to moderate physical activity in school class. Two of them performed twice per week sports training (Mitchell class IIB). None of them fulfilled WHO recommendations for physical activity in children [12].

The demographic and anthropometric features, as well as lung function baseline parameters of the enrolled population, are described in Table 1. No significant differences were observed between boys and girls.

### 3.2. Program Adherence and Safety

All 15 patients completed the study goal of performing more than 75% of the programmed training sessions. On average, each patient missed three training sessions (12%, range 1–5). A high compliance with respiratory home-training protocol was observed (100% of the subjects performed and registered more than 80% of the programmed home-training sessions). Overall, we experienced very good predisposition towards the training program and a very thorough completion rate.

Overall, no adverse events were reported during rehabilitation, except for minor muscle stiffness in the first week of training. ECG continuous monitoring showed no significant arrhythmias, only registering infrequent and non-perceived monotopic ventricular ectopy in two patients, already revealed at CPET. No adverse effects were reported during IMT training.

### 3.3. Respiratory Muscle Function

All participants in the study completed programmed measurements satisfactorily. Individual progression of respiratory muscle function is summarized in Table 2.

**Figure 1:**
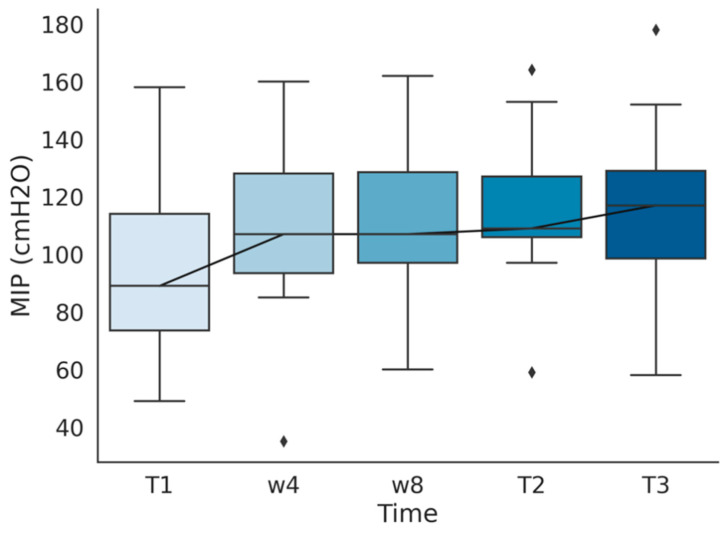
Maximum Static Inspiratory Pressure (MIP) measured before training (T1), after the first (w4) and second (w8) month of training, at the end of the program (T2), and in a six months follow-up after finishing the program (T3).

An increase in MIP (mean 94 to 116 cm H_2_O, *p* < 0.01) and percentage of predicted MIP (mean 81% to 100%, *p* < 0.01) was observed after rehabilitation. A significant increment (> 20% of predicted) was not observed more frequently in patients in a worse baseline situation (Fisher’s *p* = 0.61). This rise in MIP was maintained in a 6-month follow-up in which the subjects performed no respiratory training, observing no variation (0.5 cm H_2_O) in MIP and percentage of predicted MIP after this time. A representation of every measure of MIP performed during the program is shown in Figure 1.

On the other hand, MEP showed no statistically significant variation (mean 119 to 130 cm H_2_O, *p* = 0.12) of its absolute value or its percentage of predicted value (mean 87% to 96%, *p* = 0.11) after rehabilitation. We observed a slight increment of MEP between the end of the program and the six months follow-up (mean 130 to 138 cm H_2_O). No statistically significant difference was observed after rehabilitation in MVV (mean 80 to 86 L/min, *p* = 0.36), despite observing an increment in MVV after rehabilitation and complete stability during the follow-up period. A comparison of all measures before and after rehabilitation is represented in Table 3. Differences between measurements at the end of the program and six months after that point are shown in Table 4.

### 3.4. Functional Capacity

**Figure 2:**
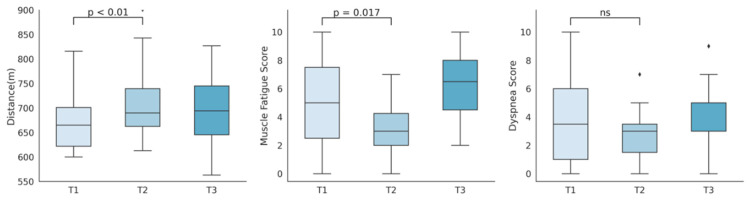
Representation of six-minute walking test distance travelled, muscle fatigue score, and dyspnea score, measured before (T1), immediately after (T2), and six months after the program (T3).

All subjects completed a valid 6MWT. Figure 2 shows the progression of distance travelled, muscle fatigue and dyspnea scales previously to rehabilitation, after training, and six months after the end of the program. Individual progression in percentage of predicted meters travelled can be found in Table 2.

A rise in the 6MWT distance travelled (m) was observed after training (mean 642 to 690, *p* = 0.001), along with a significant increase in its relation to predicted distances (92% to 99%, *p* = 0.001). This improvement was not only statistically but clinically significant, as the change of 48 m after the intervention exceeded the clinical significance threshold of 30.5 m [31]. No differences were observed between the end of the program and the six months follow-up (mean 690 to 688, *p* = 0.60). Subjects experienced a reduction in Borg muscle fatigue scales (0–10) after training (mean 4.9 to 3.2, *p* = 0.017), which presented a significant rebound at six months follow-up (mean 3.2 to 6.3, *p* = 0.0002). Although not statistically significant, the Borg dyspnea scale (0–10) showed a decrease after training (mean 3.9 to 2.8, *p* = 0.07), which again experienced a rise (mean 2.8 to 4.4, *p* = 0.03) six months after training stopped. A summary of the 6MWT distance and scales comparisons pre and post rehabilitation can be found in Table 5. In Table 6, we show the comparison between the end of the program and the six months follow-up.

## 4. Discussion

This clinical study demonstrates an improvement of MIP, distance walked, and muscle fatigue perceived in the 6MWT as surrogate measures of respiratory muscle function and functional capacity improvement, following a three-month cardiopulmonary rehabilitation program in children with congenital heart disease. In addition, its results reveal that the achieved benefits are maintained in the majority of the subjects after a period of six months following rehabilitation, being to our knowledge the first study to assess persistence of aforementioned benefits. It is arguable that a lack of statistical power due to small sample size could be potentially obscuring an actual improvement on MEP and perceived dyspnea in 6MWT, for they border statistical significance, with apparently relevant improvements.

### 4.1. Respiratory Muscle Function

Global respiratory muscle strength has been shown to be reduced in the CHD population. This ventilatory limitation has been linked to surgical scarring and thoracic deformation, phrenic nerve injury, and deconditioning, but it most certainly behaves as a multifactorial phenomenon. In our study, 46% of participants had a MIP under 80% of their predicted values, in accordance with the high described prevalence of muscle weakness in this population [11]. However, we observed that despite prior assumptions of a higher improvement chance in patients with a worse baseline situation [32,33], respiratory muscle function was improved uniformly amongst the subjects involved in the study, independently of their starting situation.

We believe that key to this finding is choosing the right intervention protocol. According to the current evidence, a threshold-type device should be used, with medium intensity workload, adjusted between 30% and 70% of the baseline MIP. An intensity inferior to 30% does not respect the principle of overload and does not modify muscle fiber structure. On the other hand, an intensity greater than 70% can cause muscle fatigue [34]. It is imperative that this load is frequently adjusted to catalyze improvement. Patients should train at least three times a week for a minimum of eight weeks. Supervision and instruction prior to home-based training is mandatory.

Paucity of studies and heterogeneity amongst intervention methodologies compromises comparability of the outcomes amongst them. Laohachai et al. proved that a six-week course of IMT muscle training for 30 min per day in adolescent/young adult Fontan patients produced a significant improvement in inspiratory muscle strength, ventilatory efficiency, and resting cardiac output [29]. In contrast, a pilot study recruited Fontan young adults, showing no improvement in MIP and MEP after IMT training for 12 weeks. However, the authors postulate that the failure to improve may be related to inadequate inspiratory load adjustment [35]. More recently, a randomized controlled trial conducted by Fritz et al. [36] recruited Fontan patients to perform IMT training daily sessions of 10–30 repetitions of IMT during six months, but the authors state that respiratory muscle function was not collected as a result. This study showed no improvement on exercise capacity or lung function. The results of our work are concordant with the data described by Laohachai et al., showing a considerable and statistically significant increase on MIP after training, with no significant changes in maximal expiratory pressure.

To our knowledge, this is the first study to evidentiate the preservation of the respiratory muscle function improvement six months after a rehabilitation program in the CHD population. These results, however, could be influenced by the follow-up period selected, as it has been previously reported in different populations that without adherence to IMT, training related gains can be lost within one year [34]. Additionally, different to previous works focused on the study of Fontan patients, we sought a more general population in order to test our hypothesis in a more representative group. This approach, however, must be interpreted carefully, as generalizing with a reduced sample makes it difficult to extract conclusions for particular cases as is later described in the limitations section.

### 4.2. Functional Capacity

The six-minute walking test represents a measure of functional capacity, integrating different physiological aspects and giving a general vision rather than the more precise assessment obtained in the cardiopulmonary exercise test. Its reliability and criterion validity have been evaluated in the pediatric population with CHD [24], showing excellent/positive criterion validity and fair agreement, despite the lack of studies to solidify this evaluation. However, its results must be interpreted and compared cautiously, as large variations of these test measurement properties exist amongst different chronic condition groups in children.

Although no minimal clinically important difference has been officially established for children in the 6MWT, previous works [24] have assumed adult values for adolescents. A systematic review conducted by Bohannon et al. [31] on adult patients with cardiorespiratory conditions establishes this difference between 14 and 30.4 m, assuming that a distance exceeding 30.5 m can be considered clinically meaningful. We find this estimation reasonable for goal-setting in this population considering our experience and results.

There is wide evidence supporting an inferior aerobic and functional capacity in the CHD population compared to healthy controls. [37]. Additionally, multiple studies have been conducted to prove the impact of CPRP in several aerobic capacity indicators. A controlled trial performed by Rhodes et al. [38] demonstrated an increase in percentage of predicted peak VO_2_ and peak work rate after a three months CPRP, as well as the preservation of the exercise function six months after completion of the rehabilitation program. More recently, a systematic review and meta-analysis by Gomes-Neto et al. [15] revealed that despite the scarcity and significant heterogeneity of publications, exercise training may improve peak VO_2_ in the CHD population, but there are no data about the repercussion on overall survival.

Most of these studies, however, focus on different outcome measures, although supporting the same general line of evidence. A study conducted by Moalla et al. [39] on the effects of training in the six-minute walking test compared CHD with control children (*n* = 17 vs. 14), and showed a reduction in baseline distance travelled of CHD children compared to healthy controls and a significant improvement in distance travelled after training. Despite differences in training protocols and supervision, we believe that our results show agreement with the data published in this study, observing a similar improvement of distance travelled, and offering a slightly larger sample (9 vs. 15 patients in the training group). Careful observation of the six months period after training in our study reveal no improvement whatsoever in 6MWT performance, compared to the mean 48 m of improvement in the three month training period, suggesting that despite lack of a formal controlled design, the same subjects experienced no spontaneous improvement over time. Additionally, our study reveals the persistence of functional capacity improvement, a new but expected outcome considering previous analysis of aerobic capacity evolution in time [35]. We found no correlation between 6MWT and respiratory muscle function, as stated in a previous investigation by Feltez et al. [40]. Concerning perceived fatigue (measured as muscle fatigue and dyspnea in our study), we found no previous evidence of the impact of exercise training on this outcome. Our results suggest an improvement of perceived leg fatigue, which has been shown to be the main exercise-limiting symptom reported by patients in some other chronic disease groups [41]. Interestingly, worsening of this score occurs after a six months period without supervised exercise training, possibly pointing out the impact of being active upon the subjective perception of fatigue.

### 4.3. Limitations

This study presents multiple limitations that could potentially affect its interpretation. Firstly, the sample size is small, in accordance with all published literature on the field of cardiac rehabilitation in children [15]. This limitation is due to the heavy time and resource requirements nature of rehabilitation programs, both for professionals and for the families. We considered it not advisable to perform rehabilitation on large groups of children, as the ability of the supervisors to guarantee correct and safe training and keep the attention of the group declines exponentially as group size grows. Additionally, the heterogeneity of the group diagnoses, combined with the aforementioned small sample size, could potentially affect the extrapolation of the results to the wide variety of CHD. This heterogeneity is caused by the sheer variation in CHD nature, and the rate at which children can be tested with CPET and screened. We believe a balance between sample size and heterogeneity must be sought. Another limitation is the lack of a control group, which could potentially affect the ascription of the effects to the intervention. This is attributable to the scarce number of patients and the difficulty for the families to attend several visits and measurements without an intervention. To counterweight this limitation, potential factors of MIP, MEP, and functional capacity improvements were discussed, and the most relevant were identified as (1) children growth and development during the three-month period and (2) the effect of training in measuring outcome. To eliminate the first, we compared the predicted values computing the weight and height at the moment the measurement was taken, accounting for the potential difference produced by mere growth. In order to minimize the effect of the latter, we thoroughly trained all children in the measurement methodology before we started data acquisition and aimed insistently for consistency in the measurements (several readings with < 10% difference). Lastly, results suggest that the statistical power of the study is limited by the sample size, and it is plausible that an impact on MEP and MVV would be observed in a larger sample.

## 5. Conclusions

In summary, we report improvements in inspiratory muscle function, functional capacity, and muscle fatigue exercise perception after a three months cardiopulmonary rehabilitation program in children with CHD. Interestingly, improvements in inspiratory muscle function and functional capacity seem to persist six months after having finished the training. These findings require validation, and further studies are clearly needed in this direction. These studies must ideally be multicentric, employ a standardized exercise protocol, and have controlled, ideally randomized design.

## Figures and Tables

**Table 1:** Demographic, anthropometric, and pulmonary function baseline characteristics of study population.

	Total (*n* = 15) Mean ± SD (range)	Boys (*n* = 9) Mean ± SD (range)	Girls (*n* = 6) Mean ± SD (range)	*p*-Value
**Demographic/anthropometric**				
Age (years)	14.4 ± 1.1 (12.4–15.7)	14.4 ± 1.3 (12.4–15.7)	14.5 ± 0.9 (13.3–15.8)	0.43
Height (cm)	161.9 ± 9.9 (143–182)	164.9 ± 10.7 (143–182)	157.4 ± 7.3 (145–165)	0.05
Body mass (kg)	52.8 ± 12.5 (33–74.2)	55.5 ± 12.9 (41.3–74.2)	48.9 ± 11.9 (33–63)	0.29
BMI (kg/m^2^)	20 ± 3.5 (14.8–25.4)	20.3 ± 3.6 (14.8–25.4)	19.5 ± 3.8 (15.7–24.3)	0.11
**Pulmonary function**				
FEV_1_ (L)	2.29 ± 0.54 (1.26–3.53)	2.34 ± 0.69 (1.26–3.53)	2.23 ± 0.23 (1.89–2.44)	0.43
Predicted FEV_1_ (%)	0.77 ± 0.15 (0.38–0.93)	0.72 ± 0.17 (0.38–0.90)	0.85 ± 0.09 (0.71–0.93)	0.05
FVC (L)	2.81 ± 0.72 (1.64–4.15)	2.93 ± 0.88 (1.64–4.15)	2.64 ± 0.37 (2.12–3.09)	0.30
Predicted FVC (%)	0.80 ± 0.17 (0.40–1.03)	0.75 ± 0.20 (0.40–1.03)	0.86 ± 0.10 (0.72–1.03)	0.10
FEV_1_/FVC ratio (%)	81.84 ± 5.98 (72.0–92.6)	79.86 ± 5.24 (72.00–88.00)	84.82 ± 6.20 (77.00–92.60)	0.06

Abbreviations: BMI = Body Mass Index; FEV_1_ = Forced Expiratory Volume in the 1^st^ second; FVC = Forced Vital Capacity.

**Table 2:** Percentage of predicted MIP, MEP, and 6MWT distance of every subject measured before, after, and six months after completion of the training program.

Subject	MIP (% Predicted)			MEP (% Predicted)			6MWT (% Predicted)		
	T1	T2	T3	T1	T2	T3	T1	T2	T3
**1**	40	49	48	70	76	70	53	75	80
**2**	94	102	100	75	97	102	49	60	62
**3**	136	112	124	111	106	104	96	99	100
**4**	129	158	172	121	100	123	98	101	109
**5**	69	82	93	79	101	114	115	119	117
**6**	87	123	109	70	79	79	94	96	94
**7**	100	95	108	105	116	121	90	101	97
**8**	56	71	67	76	50	66	93	97	101
**9**	59	98	107	69	83	112	88	90	93
**10**	91	133	129	81	114	102	99	101	102
**11**	52	88	86	52	67	70	99	109	116
**12**	82	111	100	75	125	107	103	102	102
**13**	57	93	87	107	98	91	105	104	104
**14**	72	77	68	82	79	94	96	124	104
**15**	92	154	114	129	138	150	109	109	107

Abbreviations: MIP = Maximum static Inspiratory Pressure; MEP = Maximum static Expiratory Pressure; 6MWT = six-minute walking test. T1: before training; T2: after training; T3: six months follow-up.

**Table 3:** Comparison of MIP, MEP, and MVV before and after the program (*n* = 15). Expressed as absolute and relative (percentage of predicted values) values.

	Before Training	After Training	Change (%)	Mean Difference	*p*-Value
MIP (cm H_2_O)	94.3 ± 30.1	116.1 ± 24.6	23.1	21.8	**0.001**
Predicted MIP (%)	81.4 ± 0.2	100.1 ± 0.3	23	18.7	**0.001**
MEP (cm H_2_O)	119.3 ± 32.3	130.3 ± 31.4	9.2	11	0.12
Predicted MEP (%)	87.3 ± 0.2	95.9 ± 0.2	9.8	8.6	0.11
MVV (L/min)	80.2 ± 19	85.7 ± 18.2	6.8	5.5	0.36

Abbreviations: MIP = Maximum static Inspiratory Pressure; MEP = Maximum static Expiratory Pressure; MVV = Maximum Voluntary Ventilation. *p*-values marked in bold indicate numbers that are significant on a 95% confidence limit.

**Table 4:** Comparison of MIP, MEP, and MVV immediately after the program and six months after completion (*n* = 15). Expressed as absolute and relative (percentage of predicted values) values.

	After Training	Follow-Up	Change (%)	Mean Difference	*p*-Value
MIP (cmH_2_O)	116.1 ± 24.6	116.6 ± 28.7	0.4	0.5	0.86
Predicted MIP (%)	100.1 ± 0.3	101.2 ± 0.3	1.1	1.1	0.88
MEP (cmH_2_O)	130.3 ± 31.4	137.7 ± 33.7	5.7	7.4	0.12
Predicted MEP (%)	95.9 ± 0.2	100.8 ± 0.2	5.1	4.9	0.16
MVV (L/min)	85.7 ± 18.2	85.9 ± 17.4	0.2	0.2	0.48

Abbreviations: MIP = Maximum static Inspiratory Pressure; MEP = Maximum static Expiratory Pressure; MVV = Maximum Voluntary Ventilation.

**Table 5:** Comparison of 6MWT distance, dyspnea score, and muscle fatigue score, before and after the program (*n* = 15). Expressed as absolute and relative (percentage of predicted values) values.

	Before Training	After Training	Change (%)	Mean Difference	*p*-Value
6MWT distance (m)	642 ± 128	690 ± 115	7	48	**0.001**
Predicted 6MWT distance (%)	92.5 ± 0.2	99.2 ± 0.1	7.2	6.5	**0.001**
Dyspnea after 6MWT (0–10)	3.9 ± 3.3	2.8 ± 1.9	28	1.1	0.07
Muscle fatigue after 6MWT (0–10)	4.9 ± 3.1	3.2 ± 1.9	35	1.7	**0.017**

Abbreviatures: 6MWT = six-minute walking test; Considerations: All Dyspnea and Muscle Fatigues scores at rest were 0. *p*-values marked in bold indicate numbers that are significant on a 95% confidence limit.

**Table 6:** Comparison of 6MWT distance, dyspnea score, and muscle fatigue score immediately after the program and six months after completion (*n* = 15). Expressed as absolute and relative (percentage of predicted values) values.

	After Training	Follow-Up	Change (%)	Mean Difference	*p*-Value
6MWT distance (m)	690 ± 115	688 ± 98	−0,9	−2	0.60
Predicted 6MWT distance (%)	99.2 ± 0.1	99.1 ± 0.1	−1	−0.1	0.61
Dyspnea after 6MWT (0–10)	2.8 ± 1.9	4.4 ± 2.1	16	1.6	**0.03**
Muscle fatigue after 6MWT (0–10)	3.2 ± 1.9	6.3 ± 2.3	20	3.1	**0.0002**

Abbreviatures: 6MWT= six-minute walking test. *p*-values marked in bold indicate numbers that are significant on a 95% confidence limit.

## Abbreviations and Acronyms

CHD = Congenital heart disease; CPET = Cardiopulmonary exercise test; CPRP = Cardiopulmonary rehabilitation program; HRQoL = Health-related quality of life; IMPROVE = Initiative for Monitored Pediatric Rehabilitation Outlined by Exercise testing. 6MWT = Six-minute walking test.
